# Unlicensed Practitioners

**Published:** 1887-07-02

**Authors:** 


### Unlicensed Practitioners.

*From the* Spectator, *June 18th*, 1887.

We print the following article as expressing the unprejudiced opinions of a non-professional journal on an important social question :—

Nothing could be more characteristically English than the way in which Parliament has dealt with the abuses arising from the practice of unqualified medical practitioners. It has not, like the Governments of almost all foreign countries, forbidden such persons to practise at all —it has simply made them unable to claim rewards for their services as debts. Subject to this disability, and to the risk of performing all operations and administering all drugs at his peril—that is, being liable to actions at law on account of ignorance or want of skill — the unqualified medical practitioner may put a brass plate on his door, or light a red lamp, where and when he pleases. Though at first sight it might seem as if the compromise was a dangerously lax one, it has been found in practice to limit pretty satisfactorily the number of unqualified practitioners. It is not to be regretted, however, that a Divisional Court, consisting of the Lord Chief Justice of England and Mr. Justice Denman, has lately refused in any way to extend the licence to practise medicine and surgery at present accorded in England, to persons unqualified by a regular education in medicine.

The point raised in the recent case of "Howarth *v.* Brearley" was a curious one. A certain qualified practitioner, named Fitzmaurice, employed an unqualified assistant. This unqualified assistant attended a patient for some time. When, however, the bill for this attendance was sent in, the patient refused to pay, on the ground that the assistant was not qualified, and that therefore he had incurred no liability to pay the bill. It was contended on behalf of the claimants of the money, that the debt was a debt due to the qualified practitioner, Fitzmaurice. No doubt there was, speaking technically, a good deal to be said for this contention, for the Act does not in any way forbid qualified practitioners employing unqualified assistants. Still, the assistant in the particular case had sole charge of the patient, and had prescribed for him and attended him without Fitzmaurice appearing at all in the business. Not only on this ground, but moved by general considerations, the Court decided that there was no liability. The judgment clearly shows that the Courts intended to carry out the spirit of the Act, and to interpret its provisions widely in restriction of the practice of unqualified persons in medicine.

A case like the one we are now discussing will perhaps cause our readers to consider some of the curious anomalies that exist in regard to the professions. No doubt if we go by pure reason, the regulation of the professions by law and by the bonds of professional etiquette seems utterly ridiculous. It may be argued,—Why should not the man who wants to be cured and the man who wants to cure come together on their own terms, and in any manner they like, without the interference of any third parties ? We do not prevent people injuring themselves by over-exertion or by over-drugging ; why should we hinder them from employing anyone whom they fancy can help them ? But surely even the most extreme advocate of *laissez faire* would not really wish to carry his doctrine so far as to shelter those who trade on the misery, the fear, and the credulity of mankind ? Every one must shrink from the idea of giving the shelter of the law to the quack doctor who kills his patients with his nostrums. It will hardly be contended that as the patient took the medicine voluntarily, no one has any reason to complain.

The public has the right to say :—" We cannot investigate the history and training of the doctor every time we have to call one in. Ten to one we want the doctor in a hurry, and there is no time for such investigations. We want doctors to bear a stamp of reliability, like the coinage, so that we can always feel sure we get at least a certain standard of efficiency." Now, the only way in which this standard can be kept up, is either by absolutely forbidding all uncertificated persons to practise medicine or surgery, or else by the drawing of a definite line between the qualified and the unqualified, and by the enforcing of a strict professional etiquette among the members of the qualified body. People are often unreasonably inclined to abuse professional etiquette as if it were mere nonsense. Yet it is the public who really benefit by the etiquette, and not the doctors. It is very much to the interest of the public that the medical profession should be essentially a gentleman's profession, one in which a man will not be obliged to do things repugnant to gentlemanly feeling. But without professional etiquette this would be often impossible. If it were once allowable for doctors to advertise themselves, every doctor would have to advertise himself, or fall out of the ranks. Given keen competition, and no artificial restraints on a man's power to push himself by advertisement, and we should of necessity have the humiliating spectacle of men bidding against each other in promises to relieve human suffering and to save life. There is nothing disgusting or demoralising in a man offering to sell stoves that " save half the coal," or trousers that never "bag" at the knee ; but there is something utterly disgusting in the idea of a doctor publicly offering to treat incurable diseases better than his fellows, or promising cures in an impossible time. Nothing is more demoralising than that those unfortunate enough to suffer from some deadly complaint should be at the mercy of such appeals. The struggle to keep hold of life, and the passion of feeling such a struggle arouses, make the temptation to profit thereby peculiarly dangerous and horrible. The ordinary appeals of quack-doctors and of vendors of patent medicines are bad enough ; but what would be the result if competition were to force, as it would force, the whole medical world into such appeals ? The men of fine feeling and perfect honour who now abound in the profession would be obliged to abandon a calling that would carry with it the necessity for adopting such courses as these. Yet another way in which the public benefits by professional etiquette is in the matter of fees. Suppose fees were not fixed, and that the skill of the great doctors and surgeons was, as it were, put up to auction. The poorer classes would then be unable to obtain the best medical advice as they do now. The four greatest doctors and surgeons in London might, if they liked, charge their patients ten or twenty guineas for a five minutes' consultation, and yet nevertheless have their waiting-rooms almost as crowded as they are now. The existence of professional etiquette, however, practically prevents such charges, and so confers a great benefit on the public. The strict rules as to one doctor encroaching on another's patients are also, though they sometimes seem absurd, really in the interests of the public. But for them, how would it be possible to prevent competition from assuming the most unpleasant forms ?

On the whole, it must appear, on any impartial examination of the subject, that the regulation of the professions by law, and by etiquette and custom, is indispensable. The law ought most certainly to look kindly only on those who are duly qualified (to use the phrase of Lord Ellenborough quoted by Lord Coleridge) " to make our stomachs the arena for the struggles of opposing poisons." We must give the men who drug us and cut us up immunity from prosecution in case of accidents, for if we do not, they will never dare to give any potent drug or perform any dangerous operation. In exchange, we should be most unwise not to demand that those enjoying that immunity shall be persons who are duly qualified by education to perform the duties they undertake.

